# Transactions of the Pathological Society of Philadelphia

**Published:** 1840-02-29

**Authors:** 


### TRANSACTIONS OF THE PATHOLOGICAL SOCIETY OF PHILADELPHIA.

*February 24th*, 1840.

The President, Dr. Gerhard, in the Chair.

*Case of Monstrosity, consisting in an anomalous division of the small intestine; with specimen.* By Alfred Stillé, M. D.

The child which forms the subject of this notice, was born in the lying-in ward of the Pennsylvania Hospital, of which Prof. Hodge is, at present, the attending physician.

The mother, æt. thirty, was born near Colnar, in France, and had recently arrived in this country. She, as well as her husband, are both perfectly well formed, and of good constitution. The present labour was her fourth; the previous ones had all taken place at term, and were not attended by any accident. Only one of her children, the eldest, survives: the others died; the one a few weeks after birth,—the other at the age of fifteen months, of whooping cough. During her last pregnancy, nothing whatever occurred to distinguish it from her previous ones, and labour came on within a few days of the period at which it was expected. This labour, which took place on Saturday, January 25th, was, in every respect, natural,—only, when the head had emerged from the vulva, the pains ceased for a few moments, and the delivery of the body was chiefly accomplished by the voluntary efforts of the mother. The child was a female, perfectly well formed, and active; a few hours after its birth it breathed freely and regularly, and took the breast without difficulty. On the following morning there had been no passage from its bowels; it was occasionally restless, and vomited at intervals a slimy, greenish matter, in small quantities. A teaspoonful of castor oil was administered by the mouth, when the vomiting ceased, and did not return again until the evening of the same day, when it again commenced. Nothing had passed from the bowels. A similar dose of castor oil was then given, but was soon rejected along with a considerable quantity of glairy, yellowish green matter. On the next morning, the same state of things existing, I examined the anus and rectum, and found the former well formed, and the latter readily admitting the little finger for the distance of an inch or more. The abdomen was round, hard, and resonant; there was no tumour in either groin, at the umbilicus, or elsewhere. Dr. Hodge was then consulted, and advised the use of purgative enemata, of which several were administered; the first one, composed of five ounces of fluid, was retained for several hours; the second for an hour, and the succeeding ones came speedily away. With the first one was discharged a rounded body, about eight inches long, looking very much like a portion of intestine half digested. It was evidently tubular, and marked by occasional transverse depressions; in certain portions of it, fibres like those of muscles, could be distinctly traced. It contained within a portion of its cavity an olive green substance, of pasty consistence, which, when thrown into alcohol, rapidly hardened. One or two smaller pieces of less determinate form than the above, were discharged in the course of the following days.

About the fourth day from its birth, the child refused the breast altogether, and was fed for a day or two on gum water, and afterwards on milk and water; but, at the end of a week, it could no longer take this nourishment. During this time the vomiting increased in frequency and quantity, and the matter rejected had all the physical qualities of meconium, with the odour of fœces. The child did not appear to suffer very constant pain; at times it would shriek, or cry for a few minutes, and then fall into a seemingly calm slumber; at others it would knit its brow, and contort the muscles of its face, without uttering any cry. By degrees it fell into a state of marasmus, which increased till at length its features offered the expression of the decrepitude of old age, and on the eleventh day from its birth it died with a slight convulsion.

After death, an examination was made of the body, when the organs of the chest, and those of the abdomen, were all found with their usual external characters, except the small intestine. The cavity of the abdomen was perfectly dry; but the opposite surfaces of the peritoneum adhered to one another by a sort of agglutination, of moderate firmness, but without the intervention of any false membrane. The vessels of the peritoneum were of a bright red colour, and their minute ramifications were very distinct. At about three feet from the pylorus, the jejunum suddenly terminated in a *cul de sac*, as perfect as that of the *caput coli* in ordinary cases, and which was perfectly smooth and regular, without the slightest mark of an opening or of a cicatrix. This upper part of the intestine was much distended with gas, and with a fluid similar to that which the child had vomited, and measured about an inch in diameter. At about an inch from the abrupt end of the upper division of the intestine, began the lower portion of it, which, after the usual number of convolutions, communicated with the large intestine, in the right iliac region, and through the large intestine with the anus, according to the ordinary succession of these parts. But all this lower portion of the intestinal tube was as much contracted as the upper was distended, and was no where more than a quarter of an inch in diameter. It was every where permeable. The superior extremity of it, lying near the *cul de sac* of the jejunum, looked as if it had sloughed from some contiguous part; it was almost black, pulpy, and a probe (passed into the intestine an inch below it) did not pass out through the free end, when urged against it with moderate force. The mesentery was attached to both the upper and lower divisions of the intestine, and passed from one to the other, not in a straight line, but in an arch, whose summit was attached to the origin of the mesentery at the spine.

After a careful examination of the works to which I have access, I have not been able to find the details of any case resembling that presented to the society. But several of the more recent authors who have written upon "monstrosity" in general, mention the variety of anomalous formation described above. Thus,

St. Hilaire, in his "*Hist. des Anomalies*, part ii, liv. iv, ch. v," when speaking of anomalies by division, describes a group entitled "unsymmetrical divisions of a canal producing an interruption of its cavity." "The intestinal tube," he remarks, "being composed of several sections which are developed independently of one another, is, of all the canals, the one most frequently interrupted in its course by divisions of this nature. Thus, the pharynx is, in certain cases, not continuous with the œsophagus, and each ends in a *cul de sac* at the place where their cavities ought to communicate with one another. The intestinal canal is sometimes divided into two portions, by means of a separation at the origin of the large intestine." This author does not, however, mention the case of division in the course of the small intestine; and still less does he advert to any fact like the present, where the upper division ends in a *cul de sac;* and the commencement of the lower seems to have been the seat of disease followed by sloughing of the part.

Meckel, in his "*Manuel d'Anatomie*," when describing the anormal conformations of the alimentary canal, says that "sometimes the rectum is closed only by a fine membrane, at others a solid cord or cellular tissue may supply its place. Then the large intestine may be imperforate. More rarely the small and large intestines each terminate in a *cul de sac* at their adjacent points, and yet more rarely the small intestine presents a similar interruption."

Andral thus defines the second class of occlusion of the natural openings of the intestines. "It comprehends the cases where there is not only interruption of the cavity of the intestine, but even of its walls. Thus the œsophagus has been seen completely separated from the stomach, this latter from the duodenum, and the colon from the rectum. The small intestine too has been observed to terminate in a *cul de sac* at a certain point, and the lower portion, commencing a little further on, likewise by a *cul de sac*, and then becoming continuous with the large intestine in the ordinary manner."

The only difficulty in the way of referring the malformation in the present case to the class of anomalies described in the above quotations, consists in the state of the commencement of the lower division of the intestine. The colour, the consistence, the form of that part, prove that it must have separated from some other part, and that at no very remote period from the death of the infant. From what part had it separated? Certainly not from the upper division of the intestine; for its lower extremity offered no sign of disease either recent or occurring at some distant period; its surface was smooth, polished, and without cicatrix, and had every appearance of an original formation. On the third day after the child's birth there was voided by the anus, a tubular mass which bore the closest resemblance to a portion of intestine; from the commencement of the lower division of the alimentary canal to the anus there was not the slightest departure from the common rule of formation; the portion of intestine discharged must therefore have once been connected with the upper end of the lower division, and have been separated from it by sloughing. The ordinary internal cause of sloughing of the intestine is introsusception, and this we believe to have been operative here. We suppose that the peristaltic motion having been excited either by the presence of a small quantity of meconium in the lower part of the small intestine, or by the purgatives and enemata which were administered to the child, or by some other cause, that the lower *cul de sac* was introverted, then strangulated, then detached by sloughing, and finally discharged.

Such, it would appear, is the theory to which the facts of the case naturally lead, and the only one which is at all admissible. It is to be regretted that the scientific records I have been able to examine contain no account of any parallel case. There are histories of monsters in abundance, but almost always of those which presented some deformity of the external organs so striking as to attract the notice, and excite the curiosity, of even the most negligent observers. If the bodies of children dying soon after birth were more frequently dissected, we should perhaps add something to our knowledge of the causes of death, and not be obliged, in cases like the present, almost to doubt the evidence of our senses, because the phenomena we observe are unique or rare.

